# Delta—fast ripple coupling suppression: designing a brain-mimetic stimulation paradigm for seizure abolishment

**DOI:** 10.3389/fnins.2025.1619278

**Published:** 2025-06-24

**Authors:** Uilki Tufa, Joshua A. Dian, Anya Zahra, Chiping Wu, Liang Zhang, Peter L. Carlen, Berj L. Bardakjian

**Affiliations:** ^1^Institute of Biomedical Engineering, University of Toronto, Toronto, ON, Canada; ^2^Krembil Research Institute, University Health Network, Toronto, ON, Canada; ^3^Edward S. Rogers Sr. Department of Electrical & Computer Engineering, University of Toronto, Toronto, ON, Canada; ^4^Department of Medicine, University of Toronto, Toronto, ON, Canada; ^5^Department of Neurology, University of Toronto, Toronto, ON, Canada

**Keywords:** epilepsy, deep brain stimulation (DBS), brain-mimetic, phase-amplitude coupling, thalamic stimulation

## Abstract

Deep brain stimulation can be an effective alternative treatment for patients that are intractable to antiseizure medication and do not meet surgical inclusion criteria. Clinical trials have demonstrated the safety of thalamic stimulation using a high frequency stimulus but with limited efficacy. Our group has previously shown, *in silico,* the success of stimulation with a brain-mimetic therapeutic poly-rhythmic signal, outperforming mono-rhythmic waveforms. In this study we extend our findings to an *in vivo* model and investigate a thalamic continuous stimulation paradigm using a brain-mimetic signal, where the amplitude of a high frequency rhythm is modulated by the phase of a low frequency rhythm forming a phase-amplitude coupled (PAC) waveform, to suppress seizure-like events (SLEs) in a hippocampal-kindled mouse model. We aim to show that application of our proposed “Dithered Effective Phase-Amplitude Coupled Electrical Rhythmic Stimulation (DEPACERS)” is more effective in seizure control than mono-rhythmic stimulation. Bipolar electrodes were implanted in the CA3 of the hippocampus and in the contralateral medial dorsal nucleus of the thalamus, allowing for stimulation and iEEG recordings. Video analysis was used for assessment of animal motor behavior. Mice were kindled daily through unilateral CA3 stimulations reaching evoked convulsive SLEs, then spontaneous recurrent seizures. To test suppression in fully kindled mice, thalamic stimulation using a PAC waveform was applied continuously for 15 min, followed by hippocampal stimulation to evoke an SLE. We found a 1 Hz–100 Hz phase-amplitude PAC waveform to be effective in suppressing SLEs (confirmed by iEEG and video analysis) and increasing kindling threshold. Low frequency and interictal spike suppression following interictal stimulus administration was found as a marker to assess the effective stimulus parameters. DEPACERS outperformed mono-rhythmic stimuli in evoked SLEs. These findings are important in the development of novel brain stimulation strategies for epileptic patients.

## 1 Introduction

Epilepsy is a common and debilitating neurological disorder that affects 1% of the population worldwide (Beghi, 2020). Approximately one-third of all patients have intractable epilepsies and are non-responsive to anti-seizure medications (ASMs) (Kwan and Brodie, 2000). Surgical intervention for these patients is generally a last resort attempt at controlling their seizures; however, surgical intervention is only possible in 25–50% of patients and seizure freedom following surgical intervention occurs only in 50–67% of patients (Jobst and Cascino, 2015; Samanta et al., 2021). Many factors play a role in surgical eligibility including etiology, location of seizure foci and neurocognitive testing. Neurostimulation can be an alternative treatment for patients with intractable epilepsies who do not meet surgical criteria, as well as for patients who have undergone surgery and require post-operative ASM therapy (Li and Cook, 2018; Salanova, 2018; Lozano, 2019). Clinical trials have demonstrated the safety of deep brain stimulation (DBS) using low frequency stimulation (LFS) and high frequency stimulation (HFS) with promising but limited efficacy (Salanova et al., 2021; Koubeissi et al., 2022).

DBS has been increasingly used to treat refractory epilepsy with different stimulation targets, most frequently the anterior nucleus of the thalamus (ANT) and the centromedian nucleus of the thalamus (CM) (Koubeissi et al., 2022; Schulze-Bonhage, 2017; Cukiert and Lehtimäki, 2017). Unlike responsive neurostimulation (RNS) that is applied in a closed-loop manner directly to seizure foci, DBS is open-loop and continuous, targeting non-specific nuclei that have generalized effects on cortical excitability and disrupt the thalamo-cortical network known to be involved in seizure generation (Schulze-Bonhage, 2017; Warsi et al., 2022). Thalamic stimulation is delivered using high-frequency periodic square-pulsed waveforms with varying pulse widths and cycles as both intermittent and continuous stimulation are used in different epilepsy centers (Cukiert and Lehtimäki, 2017; McIntyre et al., 2015). Initial median seizure suppression rates of approximately 60% have been observed with deep brain stimulation of the ANT or CM (Salanova et al., 2021; Warsi et al., 2022). These rates tend to increase over time when accounting for patient dropout. Despite the promise of this treatment, widely varying stimulation parameters and few patients achieving seizure freedom make current reported deep brain stimulation paradigms suboptimal treatments for epilepsy. In this study we aim to explore a novel brain-mimetic stimulus waveform termed Dithered Effective Phase-Amplitude Coupled Electrical Rhythmic Stimulation (DEPACERS) in a kindled mouse model of epilepsy.

There are three main factors that contribute to the success of a stimulation paradigm: the timing of stimulation, the location of stimulation, and the stimulus waveform. The temporal application of the stimulus is a major factor in DBS effectiveness. In contrast to responsive neural stimulation that relies on a seizure detection algorithms to apply the stimulation when a seizure is detected, open-loop approaches are generally employed with deep brain stimulation (Schulze-Bonhage, 2017). Closed-loop systems are ideal when attempting to compare stimulus parameters; however, they are dependent on the efficacy of the detection algorithm. We used an evoked kindled model to elicit seizure-like events on demand. This eliminated the need for an accurate seizure prediction algorithm, controlled the timing variable, and mimicked a closed-loop system. The location of the stimulation was chosen to be the medial dorsal thalamus as it has been shown to be associated with both the initiation and propagation of seizures (Zhang and Bertram, 2015; Bertram et al., 2008; Zumsteg et al., 2006). In addition, activation of mesial temporal areas has been observed following stimulation of the medial dorsal thalamus. The traditional DBS waveform comprises square biphasic pulses with a stimulation frequency above 80 hertz. We sought to use a brain-mimetic stimulation paradigms to improve the efficacy and responsiveness to electrical stimulation. Adapting the stimulus amplitude to underlying conditions remains a key factor in a successful brain-mimetic stimulation paradigm (Brocker et al., 2017; Grill, 2018). In addition, electrical rhythms of the brain contain non-linear coupling between low and high frequencies. Hence, we integrate these features in our DEPACER stimulation.

Our group has previously shown, *in silico,* the success of stimulation with a brain-mimetic therapeutic signal, outperforming mono-rhythmic periodic waveforms such as LFS and HFS (Zalay and Bardakjian, 2013). In this study we extend our findings to an *in vivo* rodent model and investigate a thalamic continuous stimulation paradigm using the DEPACER stimulus, containing two rhythms where the amplitude of a high frequency rhythm is modulated by the phase of a low frequency rhythm forming a PAC waveform, to suppress seizures in a kindled mouse model. We aim to show that application of our brain-mimetic stimulation is more effective in seizure control than periodic stimulation.at presentation, physical exams and lab results.

## 2 Methods

### 2.1 Animal and surgical procedure

Male C57BL/6 N mice (C57BL/6 N; Charles River Lab, Saint-Constant, Quebec) were housed with food and water ad libitum in a vivarium at 22–23° C with a 12-h light on/off cycle (light-on 6:00 am). We kindled middle-aged mice (11–13 months) to model TLE in adult populations. All experiments conducted in this study were approved by the Animal Care Committee of the University Health Network according to the guidelines of the Canadian Council on Animal Care. All surgeries were conducted under isofluorane anesthesia and a stereotaxic frame with two micromanipulators for placement of two pairs of twisted-wire bipolar electrodes. Electrodes were constructed as previously described (Bin et al., 2017; Song et al., 2018; Liu et al., 2021). All electrodes were made of polyamide-insulated stainless steel wires (100 or 230 μm outer diameter; Plastics One, Ranoake, VA). Wires with 100 μm diameter were used in most of experiments to minimize electrode-related tissue injury/perturbations. Twisted bipolar electrodes were used for stimulation and recording. For local differential recordings, the two open ends of each implanted bipolar electrode were connected to two inputs of an amplifier headstage. One pair was positioned in the hippocampal CA3 region for kindling stimulation and recordings, and the other was placed in the contralateral dorsal-medial thalamus (bregma −1.5 mm, lateral 0.5 mm and depth 3.5 mm). A reference electrode was situated in a frontal area (bregma +1.5 mm, lateral 1.0 mm and depth 0.5 mm). The electrode locations were later verified with histological assessments as previously shown (Liu et al., 2021).

### 2.2 Hippocampal kindling and thalamic stimulation

**Figure 1:**
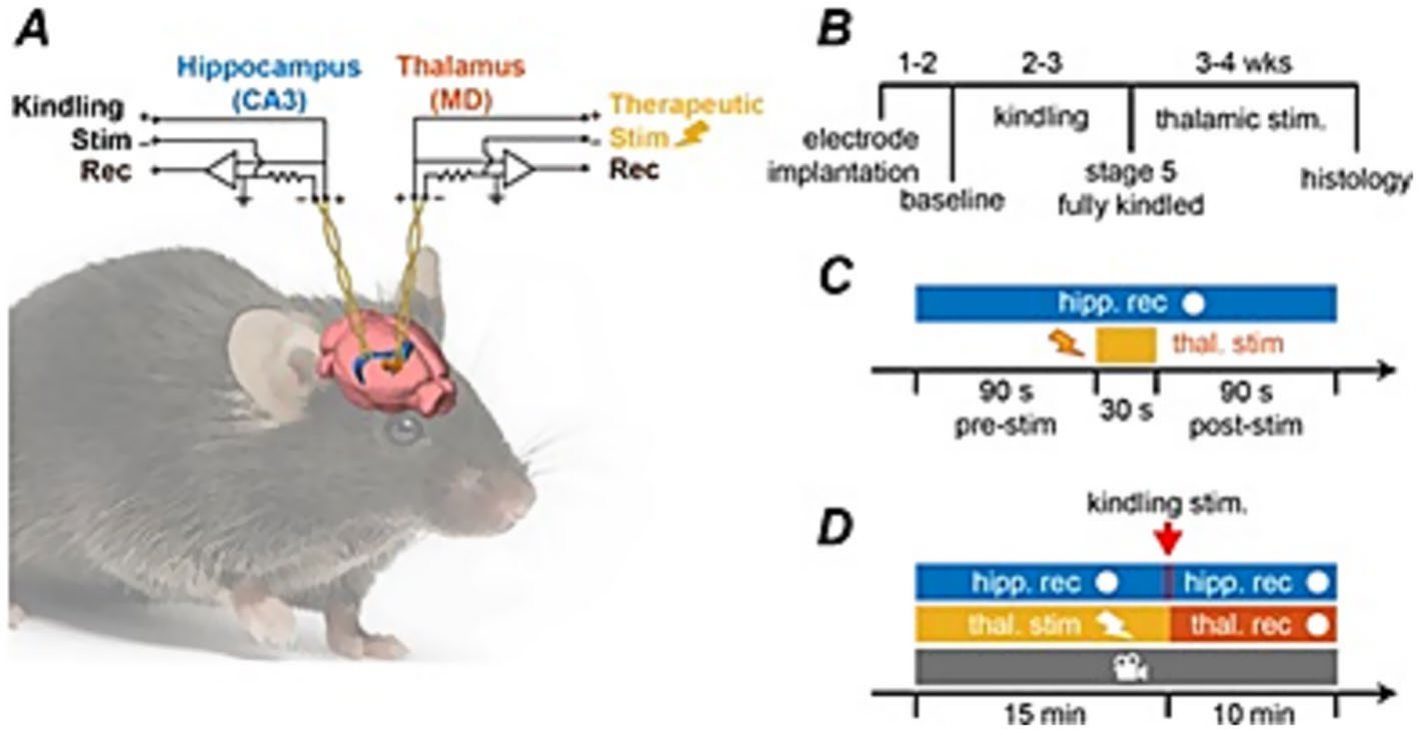
Schematic of experimental design. **(A)** Bipolar electrodes implanted in the CA3 of the hippocampus and the contralateral medial dorsal thalamus. Experimental timeline showing: **(B)** the kindling process with thalamic stimulation experiments performed after animals reached the fully kindled state exhibiting stage 5 convulsive seizures, **(C)** short thalamic stimulation for amplitude assessment, **(D)** thalamic stimulation applied prior to a hippocampal kindling stimulus.

For hippocampal kindling, a train of stimuli at 60 Hz for 2 s was utilized (Bin et al., 2017; Liu et al., 2021; Reddy and Rogawski, 2010; Jeffrey et al., 2014; Stover et al., 2017). A Grass stimulator was used to generate constant current pulses with monophasic square waveforms, 0.5 ms pulse duration and current intensities of 10–150 μA, delivered through a photoelectric isolation unit (model S88, Grass Medical Instruments, Warwick, Rhode Island, United States). An ascending series was executed to determine the threshold of evoked after-discharges (ADs) in individual mice, which was then used for kindling. Kindling stimuli were applied twice daily and at least 5 h apart (Figure 1) (Song et al., 2018; Liu et al., 2021). Each stimulation episode was monitored through video-EEG while the mouse was placed in a modified cage, and lasted for a few minutes (Song et al., 2018; Liu et al., 2021; Stover et al., 2017). Using video-EEG, kindled seizure activity was graded on the modified Racine scale.

Thalamic stimulation was delivered through bipolar electrodes implanted in the dorsal-medial thalamus. Stimulation waveforms were created using MATLAB and exported as .txt output files with a generated sampling frequency of 10 kHz. Files were then imported into a custom created Spike2 protocol and waveforms were used as output to the Digital-to-Analog (DAC) output of the Micro-1401 acquisition unit. This voltage analog output was then fed as an input to a stimulus isolation unit (A-M Systems Analog Stimulus Isolator Model 2,200) where the stimulus was delivered as current to the stimulating electrodes (Figure 1).

### 2.3 DEPACER stimulation

The stimulation waveform was generated in MATLAB using customs scripts. A cross frequency coupled signal was generated with a low frequency component ($f_{L}$) which modulated the amplitude of the high frequency rhythm ($f_{H}$). The signal amplitude was given by $A_{\text{HFR}}(t)=A_{0}*\sin(\varphi_{\text{LFR}}(t))$ where $\varphi_{\text{LFR}}$ was the phase of the low frequency rhythm. Further, the interpulse interval of the high frequency rhythm interval was drawn from a normal distribution with a mean frequency of $f_{H}$ and variance of 10 Hz providing the dithered component of the signal.

### 2.4 Data acquisition

Digitization and recording were done at a sampling rate of 10,000 Hz (Spike 2 software version 7, and Micro-1401, Cambridge Electronic Design). Signals were amplified using an A-M Systems AC/DC Differential Amplifier Model 3,000 using a low pass filter cutoff of 1 kHz and high pass filter cutoff of 0.1 and 1 Hz for thalamic and hippocampal recordings, respectively, to filter out large deflection artifacts due to monophasic kindling stimulus. A digital gate was used to switch between recording and stimulation mode that was programmed via a Spike2 acquisition protocol. Simultaneously with the LFP recording, mice were video-monitored using a webcam (Logitech) positioned in front of the recording cage to assess their movements on a modified Racine scale.

### 2.5 EEG analysis

All EEG analyses were computed using custom MATLAB scripts. Continuous wavelet transforms (CWT) were used to analyze EEG rhythms using a complex Morlet mother wavelet


Ψ(t)=1π⋅fbe2πi⋅fc⋅te−x2fb

where f_c_ and f_b_ are the central frequency and bandwidth parameters of the wavelet. A central frequency of 0.8125 Hz and bandwidth of 5 Hz was used chosen as previously used on EEG data (Grigorovsky et al., 2020). Wavelet transforms were z-scored normalized to compare effect of thalamic stimulation on brain rhythms using the mean and standard deviation of each frequency range of the wavelet transform computed prior to stimulation.

PAC strength was assessed using the algorithm by Tort et al. (2010) where phase (ϕ) and amplitude (A) of different frequency bands were obtained via the CWT and defined as

ϕ(t^,fL)=arctanIm{W(t^,fL)}Re{W(t^,fL)}, and A(t^,fH)=∣Re{W(t^,fH)}+jIm{W(t^,fH)}∣ respectively. The low frequency range used for the phase information was 1–32 Hz and the high frequency range used for the amplitude information was 32–512 Hz with increments on a logarithmic scale. The coupling strength was then computed as the normalized Kullback–Leibler distance of the amplitude distribution over the binned phases (20^o^ bins) from a uniform distribution. The PAC strength was computed using a sliding window of 4 s to allow for a minimum of 4 cycles per frequency band.

### 2.6 Statistical analysis

Wilcoxon signed-rank test was used for two-group comparisons of seizure duration with and without stimulation. Mean and standard error of the mean (SEM) were presented in group comparison figures, where n represents the number of mice used in each experiment. Statistical significance was set at *p* < 0.05.

## 3 Results

### 3.1 Determining stimulation parameters via interictal thalamic stimulation

**Figure 2:**
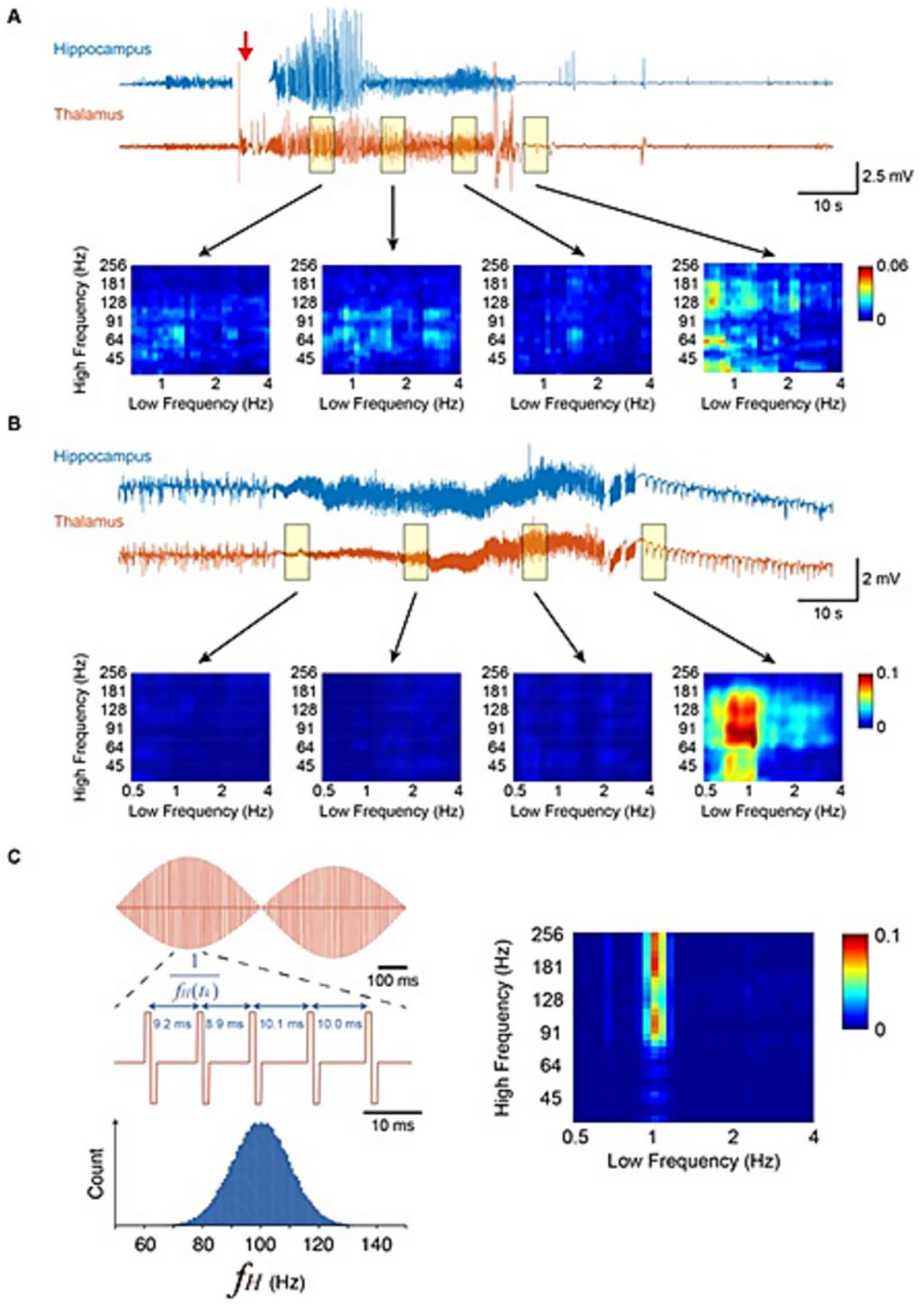
DEPACER stimulus was created using phase-amplitude coupling features found at termination of evoked and spontaneous seizures. **(A)** iEEG recordings from the hippocampus and thalamus during an evoked seizure. A kindling stimulus (red arrow) is applied to evoke a convulsive seizure. Delta-HFO PAC shown to be increased at termination in **(A,B)**. **(C)** DEPACER stimulation waveform created using phase-amplitude cross frequency coupling of 1 Hz and a range of frequencies centered around 100 Hz and following a normal distribution.

In order to successfully abolish evoked seizure in Stage 5 kindled mice, it was necessary to identify the parameters of the DEPACER stimulation including stimulation frequencies and amplitude. The frequencies used in the phase-amplitude coupling of the therapeutic thalamic stimulation were chosen based on the same frequencies found coupled together at seizure termination. The PAC strength was assessed within 4 s windows spanning different segments of the seizure in the medial dorsal nucleus of the thalamus recording (Figure 2). A delta rhythm coupled with high frequency oscillations (HFOs) was found at the termination of evoked (Figure 2A) and spontaneous recurrent seizures (Figure 2B). The stimulation paradigm was made more brain-mimetic by choosing these parameters found at seizure termination to successfully drive the system away from a seizure state (Guirgis et al., 2015; Sobayo and Mogul, 2015). The frequencies chosen in our brain-mimetic stimulation waveform consisted of 1 Hz and 100 Hz, with the phase of the low frequency modulating the amplitude of the high frequency (Figure 2C). This DEPACER output waveform contained bi-phasic square pulses of 1 ms whose amplitude followed the phase of a sinusoidal 1 Hz signal (Figure 2C). This is consistent with physiological rhythms of the brain which contain cross-frequency coupling and time varying amplitudes (Kalitzin et al., 2019).

In order to further make the stimulation waveform brain-mimetic and avoid adaptation, the high frequency component followed a Gaussian distribution with mean of 100 Hz and variance of 10 Hz (Figure 2C).

**Figure 3:**
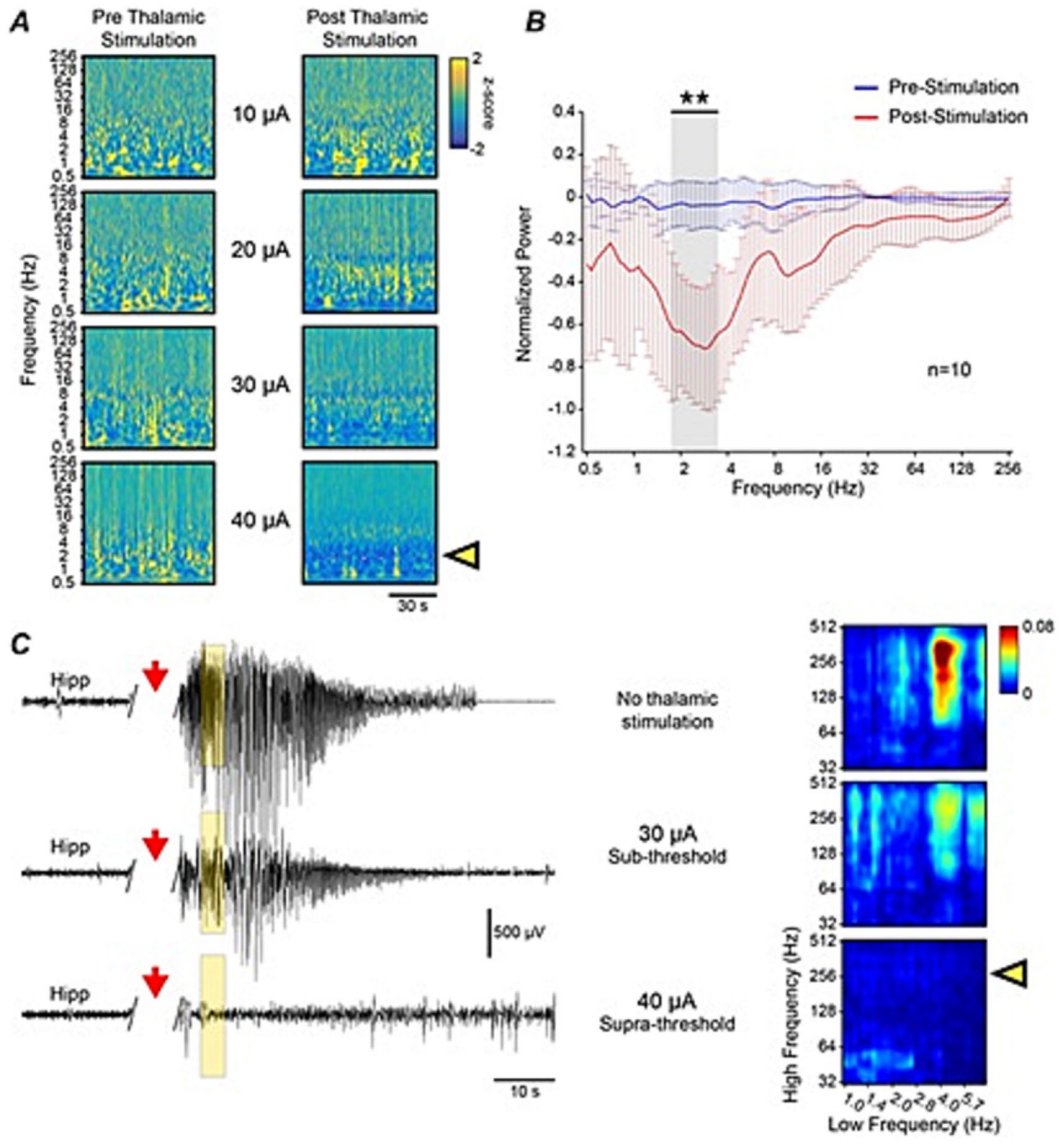
Determining successful amplitude of thalamic DEPACERS. **(A)** DEPACERS applied at increasing amplitudes (peak-to-peak) during interictal periods. Normalized wavelet transform showing a decrease in delta frequencies (yellow arrow) after administration of 40 μA thalamic stimulation. **(B)** Normalized spectral power for group of animals before (blue) and after (red) thalamic stimulation showing a significant (**) decrease in the delta range (*p* < 0.001). **(C)** Hippocampal recordings following kindled stimulus (red arrow) and different amplitudes of DEPACERS along with PAC comodulograms corresponding to highlighted windows of traces (top: no stimulation; middle: sub-threshold stimulation; bottom: supra-threshold stimulation). Yellow arrow indicates suppression of pathological PAC when supra-threshold thalamic stimulus is applied.

The amplitude of the chosen waveform was identified through an interictal excitation threshold. Electrical stimulation with the DEPACER stimulus waveform was administered for periods of 30 s in interictal periods of stage 5 kindled mice in increasing amplitude steps of 10 μA (Figure 3). The response to this stimulus was then investigated by looking at differences in spectral power through a normalized wavelet transform. Wavelet coefficients during the 30 s stimulation period were ignored due to the stimulating artifact. A threshold was identified using this approach where the amplitude of the DEPACER stimulus was sufficient to induce changes in the spectral power of the hippocampal EEG. More specifically, a suppression in the delta frequency range was identifed (Figure 3) and was used to identify the “dose–response” relationship of the brain-mimetic stimulus. Thresholds varied in individual mice and ranged from 10 to 80 μA., It was found that the DEPACER stimulus could only abolish Stage 5 evoked seizures if the stimulation amplitude reached the identified threshold (Figure 3). The DEPACER stimulus was then tested on evoked seizures using the previously identified parameters, where an interictal excitation assessment determined the amplitude.

### 3.2 Comparing DEPACER to mono-rhythmic periodic deep brain stimulation in a stage 5 kindled model

**Figure 4:**
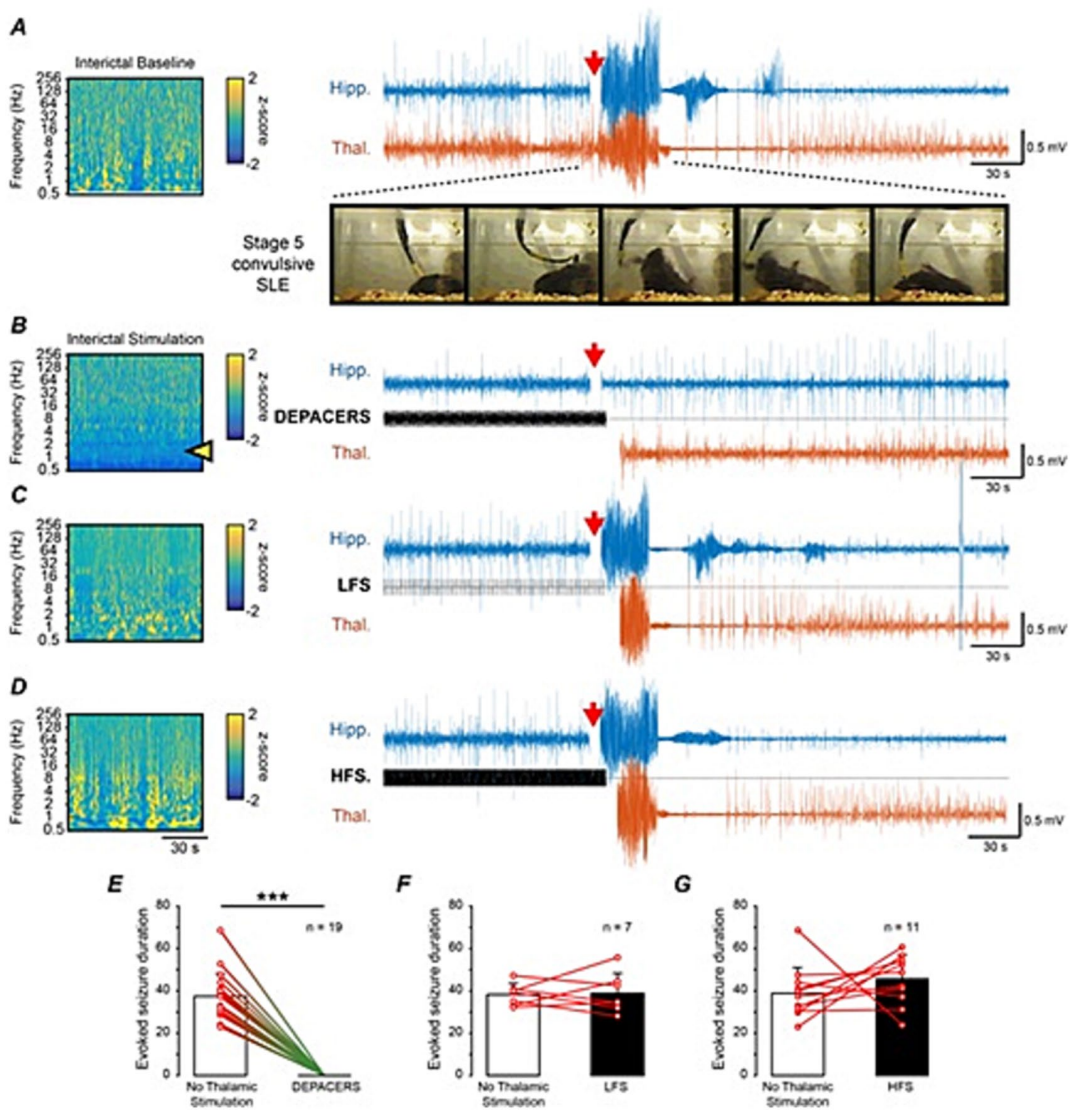
Thalamic DEPACER stimulation outperforms mono-rhythmic high frequency stimulation in suppression of evoked seizures. Normalized wavelet transform showing effect of interictal stimulation and corresponding iEEG traces of the hippocampus and thalamus during an evoked seizure with **(A)** no thalamic stimulation, **(B)** DEPACER stimulation, **(C)** mono-rhythmic LFS and **(D)** with a mono-rhythmic HFS. Thalamic stimulation was applied for 15 min prior to the evoked stimulus which is indicated by the red arrow in **(A–D)**. Evoked discharges accompanied by Stage 5 convulsive events as shown in video frames **(A)** except when suppressed by DEPACER stimulation **(B)**. Delta suppression was seen after the administration of the DEPACERS **(B)** during interictal periods but not after LFS and HFS **(C,D)**. **(E)** Evoked seizure duration showing significant (***) suppression due to thalamic DEPACER stimulation (*p* < 0.001). **(F,G)** No significant difference was seen due to thalamic LFS and HFS as compared to control (*p* > 0.05).

DEPACER stimulation administered in the contralateral thalamus was compared to mono-rhythmic periodic stimulation waveforms of 1 and 100 Hz without PAC. The amplitude of the DEPACER stimulus was found through the interictal threshold assessment as previously described. The same amplitude was used for both the low frequency stimulation of 1 Hz and the high frequency stimulation of 100 Hz for comparison. Figure 4A shows the interictal spectral power and EEG during the evoked seizure from a Stage 5 kindled mouse without thalamic stimulation. When the kindling stimulus was applied above the kindling threshold, we were able to reliably generate an evoked seizure, observed electrographically in the EEG and behaviorally through video analysis showing bilateral forelimb clonus, rearing and falling which corresponded to a stage 5 on the modified Racine scale. Figure 4B shows the interictal response to DEPACER stimulus (30 s in duration) showing suppression of the delta-range frequencies. We tested the therapeutic stimulus by applying it to the thalamus 15 min prior to the application of the kindling stimulus and extending by 6 s chosen to cover a wide therapeutic time-window (Zhong et al., 2012) to try and remove the temporal variable of its antiepileptic effect and be able to compare only the waveform. The DEPACER stimulus was able to suppress and abolish the evoked seizure (Figure 4B) as observed by the absence of an electrographic seizure in the EEG channels as well as any behavioral correlates. We also observed a suppression of interictal epileptiform discharges in the kindled hippocampus channel when the DEPACER stimulation was turned on. EEG recordings could not be obtained while the stimulus was applied, accounting for the gaps in the traces, as the specific channel was switched to stimulation mode. Figures 4C,D show the effect of mono-rhythmic low frequency and high frequency stimulation, respectively. We observed that the mono-rhythmic waveforms were not able to suppress the evoked seizures as the EEG traces showed the same discharges as the evoked seizure in Figure 4A without any therapeutic stimulation in the same animal. These seizures were also associated with modified Racine stage 5 convulsions. Comparing the therapeutic thalamic stimulation to no stimulation demonstrated that the DEPACER stimulus outperformed both 1 and 100 Hz mono-rhythmic stimuli in abolishing seizures (Figures 4E–G). The DEPACER stimulation significantly abolished seizures in all mice supported by the absence of electrographic seizures and behavioral manifestations (*n* = 19 animals, mean evoked duration 37.4 ± 12.2 without stimulation as compared to 0 ± 0 with stimulation, *p* < 0.05). The low frequency (*n* = 7 animals, mean evoked duration 38.3 ± 5.6 as compared to 39.1 ± 7.4 with stimulation, *p* > 0.05) and high frequency stimulation (*n* = 11, mean evoked duration 39.4 ± 12.7 as compared to 44.2 ± 10.9 with stimulation, *p* > 0.05) did not significantly alter the evoked seizures without thalamic stimulation (Figures 4E–G). These data strongly demonstrate that DEPACER stimulation is able to abolish seizures, outperforms mono-rhythmic stimulation and that the low frequency suppression can be used as a marker to test the efficacy of the stimulation.

## 4 Discussion

Neuromodulation has been shown to be a safe and effective method in reducing the frequency of seizures in patients who have drug-resistant epilepsy and are ineligible for surgical resection. DBS has shown mixed results, similar to other neuromodulation approaches such as vagus nerve stimulation and responsive focal stimulation, with suboptimal seizure reduction rates and some patients not achieving any improvements (Haneef and Skrehot, 2023). The 10 year follow-up of the SANTÉ trial showed a median seizure reduction of 75% from baseline; however, only two out of the 73 subjects were seizure-free for an extended period of time (Salanova et al., 2021). In this study we demonstrate a brain-mimetic stimulation paradigm for seizure abolishment.

**Figure 5:**
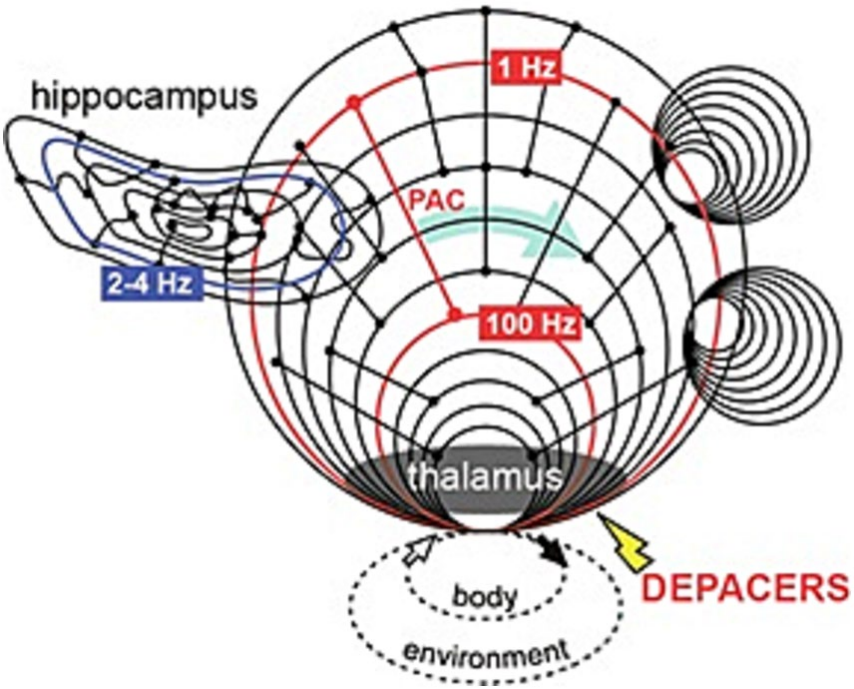
Schematic showing the effect of DEPACERS on the thalamus hub through phase-amplitude coupling of delta and HFO, causing a decrease in delta oscillations in the hippocampus and disrupting the network responsible for seizure initiation. Adapted from Haneef and Skrehot (2023) and Buzsáki (2006).

Thalamic stimulation as a therapy for epilepsy is that the treatment does not require specific knowledge of the seizure foci *a priori* as it targets brain regions that play a role in the synchronization, maintenance and propagation of seizures (Fisher and Velasco, 2014). The thalamus is an important integration hub in the brain, organized in multiple parallel loops that make it highly interconnected within the brain (Figure 5; Buzsáki, 2006). The dorsal medial nucleus of the thalamus was chosen as our target for stimulation as it is implicated both in the initiation as well as the propagation of kindled seizures and has been suggested to have a major role in seizure synchrony (Zhang and Bertram, 2015; Bertram et al., 2008; Bertram, 2014). PAC has been observed in EEG recordings and implicated in network communication and synchrony and has also been used to identify the epileptogenic zone (Guirgis et al., 2015). In this study, we explore a novel stimulation waveform termed DEPACERS that uses PAC features found at seizure termination to drive the brain state away from seizures and toward a state that resembles termination (Sobayo and Mogul, 2015; Sobayo and Mogul, 2016). In order to compare the efficacy of this brain-mimetic stimulation that involves two interacting frequencies, 1 and 100 Hz, it was important to compare the effect of each individual frequency alone. Here we explored and contrasted a brain-mimetic stimulation waveform to mono-rhythmic periodic high and low frequency stimulation waveforms that resemble the stimuli currently used clinically. Comparing the application of DEPACERS to mono-rhythmic pulses showed that the frequency components contained in the DEPACER stimulation have a synergistic rather than additive effect that fully abolishes seizures. This may be due in part to multiple proposed mechanisms being at play when the brain is stimulated using a cross-frequency coupled waveform. The DEPACER signal could be benefitting from local inactivation by activation of GABA-ergic inhibitory neurons due to its high frequency component as well as causing a long lasting hyperpolarization and inducing long term depression due to its low frequency component (Toprani and Durand, 2013). Low frequency stimulation in the range of 1–20 Hz was shown to reduce seizure frequency in rodents through a GABA_A_-mediated mechanism both *in vitro* and *in vivo* (Chiang et al., 2014; Ladas et al., 2015; Salam et al., 2015). Moreover, the interplay between the two frequency could alter or disrupt information flow, analogous to clinical effects from lesioning, as well as coupling between different brain regions that contribute to the initiation, propagation and spread of the seizure (Grill et al., 2004; Lowet et al., 2022). The low frequency suppression following interictal DEPACER stimulation supports this type of network effect as low frequencies have been suggested to modulate activity over large spatial regions. Here we have shown that the DEPACER stimulus is effecting a region that is beyond the targeted area as we are administering the stimulus in the medial dorsal nucleus of the thalamus contralateral to the kindled CA3 site in the hippocampus.

Our group has previously demonstrated algorithms for seizure prediction in humans leveraging cross-frequency coupling and machine learning (Lucasius et al., 2024; Jacobs et al., 2018). These algorithms are likely amenable to seizure prediction in animals such as those used in this study. Combining the prediction algorithm with DEPACER stimulation would allow testing a complete closed-loop seizure suppression systems and forms and application we anticipate performing in future studies.

Dithering is also an important feature of the DEPACER stimulus where the high-frequency component varies based on a normal distribution centered around 100 Hz. The successful seizure abolishment could be partly due to a higher specificity related to the dithered stimulus. The introduction of noise in dithered brain stimulation has been proposed to result in more selective entrainment (Duchet et al., 2023). Additionally, higher complexity measures and neuronal noise were observed in the interictal state as compared to ictal state (Serletis et al., 2012). The added noise and variability of the DEPACER stimulus may prevent the transition into an ictal state. Further studies need to be done to elucidate the mechanism and explore dithering parameters.

Delta oscillations play an important role in seizures, found in local field potential recordings of seizures in animal models, shown to be responsible for synchronizing multi-unit discharges both in non-convulsive and convulsive seizures (Chou et al., 2020). In a study by Colic et al., 2016 of a Mecp2-deficient model of Rett syndrome and epilepsy, 2–5 Hz delta rhythm was found as a biomarker in the prediction of antiepileptic drug treatment (Colic et al., 2016). Additionally, the delta-HFO PAC was shown to be present in recorded EEG epileptiform activity in this mouse model and was abolished with pharmacologic rescue treatment of the animal. Our finding of delta rhythm suppression by DEPACER stimulation fits with this result and shows the same phenotype. This suggests that the suppression of the delta rhythm, also suppresses the pathologic delta-HFO PAC.

These oscillations have also been observed in human intracranial EEG recordings where a delta rhythm modulating high frequency oscillations was found to be associated with seizure dynamics (Guirgis et al., 2013). The region of increased low-frequency coherence in the interictal state was similar to the region of increased high-frequency coherence in the ictal state (Cotic et al., 2015). This study supports our findings that low-frequency perturbations during interictal periods can be used to assess ictal network modulation. This result is also in line with findings by McConnell et al. showing a correlation between the effect of DBS in a rodent model of Parkinson’s disease and the suppression of pathological low-frequency oscillations in the basal ganglia via high-frequency stimulation (McConnell et al., 2012). It is possible that the low frequency suppression induced by the DEPACER stimulation prevents the emergence of the delta rhythm responsible for coordination of brain regions and initiation of seizures.

The delta frequency range suppression following thalamic PAC stimulation is an important finding as this marker can lead to adaptive stimulation parameters that may differ from patient to patient in the clinic. Currently, stimulation parameters are changed *ad hoc* by physicians based on assessing changes in seizure incidence through regular visits. Our interictal stimulation marker can eliminate the need for assessing seizure incidence and provide faster feedback in choosing stimulation parameters by probing the aberrant networks during interictal periods. Furthermore, customized programming of stimulation parameters can be done remotely through regular feedback from the neurostimulator allowing for an adaptive stimulation paradigm. This study demonstrates a clear proof of principle for a DEPACER stimulus waveform to abolish seizures and which may also serve as a neuromodulation therapy for patients at high risk for sudden unexpected death in epilepsy (SUDEP).

## 5 Conclusion

In this study, we have shown that DEPACER stimulation is more effective than LFS and HFS periodic stimulation in suppressing seizure-like events in a kindled mouse model of epilepsy. The DEPACER waveform resembled brain activity through delta-HFO PAC with dither similar to those present in EEG at seizure termination. Low-frequency suppression following short stimulation during interictal periods was used as a biomarker to guide successful and adaptive stimulation amplitude. These results are significant for the advancement of new brain stimulation techniques that could be used to treat epileptic patients.

## Data Availability

The raw data supporting the conclusions of this article will be made available by the authors, without undue reservation.
